# Grassroots circularity in Bologna: rethinking circular economy pathways for socio-ecological transition

**DOI:** 10.3389/fsoc.2026.1810660

**Published:** 2026-06-03

**Authors:** Alessandra Landi, Mattia Lucertini, Francesca Cappellaro

**Affiliations:** 1 Department of Sociology and Business Law, Alma Mater Studiorum-University of Bologna, Bologna, Italy; 2 Technology Transfer Directorate, Technology Transfer Tools, Bologna Research Centre, ENEA-Italian National Agency for New Technologies, Energy and Sustainable Economic Development, Bologna, Italy

**Keywords:** Bologna, circular economy, grassroots circularity, participatory methods, living lab, urban transition

## Abstract

The circular economy (CE) has emerged as a prominent framework for addressing environmental challenges while promoting collective well-being. By aiming to “close the loop” of product life cycles through reuse and recycling, the CE encourages entrepreneurial and economic activities oriented toward ecological transition and sustainable urban development. However, CE implementation remains predominantly technology-centric and industry-driven, privileging innovation and system optimization - approaches increasingly seen as insufficient to support transformative socio-ecological change. Consequently, CE policies often remain confined to waste management, overlooking preventive strategies and the potential of social innovation. This study investigates the contribution of grassroots initiatives to circular transitions in the metropolitan city of Bologna (Italy) and examines whether gaps in the supporting ecosystem and infrastructure hinder their implementation. The analysis focuses on the participatory project *R-innovare l’Economia Circolare*, developed within the NRRP ECOSISTER program and involving 17 organizations engaged in reuse, repair, sharing, and regeneration practices. Using participatory methodologies - including contextual mapping and facilitated co-design workshops - the study identifies actors, circular practices, and business models, as well as implementation barriers emerging at the organizational, community and institutional levels. Rather than focusing on technological optimization or recycling, findings reveal a heterogeneous set of circular practices centered on waste prevention, community engagement, and social equity, which diverge from mainstream CE models. These practices point to emerging forms of collaboration and service-based approaches that generate socio-environmental value by integrating ecological care with community well-being. At the same time, identified challenges include limited access to spaces and resources, insufficient incentives for non-technological innovation and widespread reuse practices, and a fragmented governance landscape marked by conflicting policy domains. Drawing on the Bologna case, the study shows how grassroots actors reinterpret circularity and broaden the transformative potential of the CE beyond recycling and the economic sphere, reframing it as a process rooted in care, solidarity, and territorial well-being. The analysis also highlights gaps within policy and research infrastructures that must be addressed to advance a more just and place-based socio-ecological transition.

## 1 Introduction

The transition to a circular economy (CE) is increasingly framed as both a challenge and a key opportunity for advancing socio-ecological transition (Ghisellini et al., 2016). These dynamics are particularly relevant at the urban scale, where cities are increasingly positioned as laboratories for circular innovation. Building on this context, this work explores grassroots circular practices in the metropolitan city of Bologna^1^, Italy. Drawing on a participatory living lab conducted in 2025 within the NRRP funded ECOSISTER project^2^, the paper addresses two research questions: (1) How do grassroots initiatives apply and reinterpret the circular economy in an urban context, and through which circular strategies and business models? (2) What barriers, challenges and infrastructural gaps affect their capacity to contribute to circular transitions?

The paper is structured as follows: section 2 presents the conceptual framework and key debates on the plural and contested nature of the circular economy, including its application to urban contexts; section 3 outlines the methodology and describes the *R-innovare l’Economia Circolare* (R-innovate the Circular Economy) living lab research design; section 4 reports the empirical results, including profiles of engaged organisations, mapped strategies and emerging circular business model archetypes, as well as identified infrastructural and governance barriers and needs. Section 5 discusses the findings in relation to debates on the circular economy and urban socio-ecological transitions; section 6 concludes with key contributions and future research directions.

## 2 Conceptual frameworks and key debates

Generally understood as an economic system that is restorative and regenerative by design, the CE aims at the sustainable management of resources and material stocks by keeping their value within society for as long as possible. However, rather than constituting a unified paradigm, CE has evolved into a plural and contested concept, with more than 221 definitions appearing in the literature (Kirchherr et al., 2023). Such conceptual plurality reflects that different actors articulate circularity according to their position on fundamental issues (e.g., social, technological, political, ecological). Multiple CE discourses have accordingly been identified, ranging from holistic and transformative visions to more segmented and technocentric interpretations (Calisto Friant et al., 2020). This interpretive flexibility fosters adaptability and diffusion of CE across sectors and policy arenas, but it also risks diluting its original ambitions (Corvellec et al., 2022; Murray et al., 2017), sometimes leading to unsustainable “pseudo circular” practices (Velenturf and Purnell, 2021). Recent global assessments further reveal that progress towards CE is falling short: according to the Circularity Gap Report, the share of secondary materials fed back into the global economy declined from 7.2% in 2018 to 6.9% in 2021 (Circle Economy, 2025).

In practice, CE implementation has largely been driven by industry oriented and technology centred approaches, with a strong emphasis on waste management, recycling and resource efficiency (Calisto Friant et al., 2021). Following Ruggerio (2021), this trajectory has been associated with a weak sustainability model that leaves dominant growth paradigms unchallenged. The European Environment Agency has called for moving beyond recycling toward strategies prioritising waste reduction, reuse and reduced consumption (European Environment Agency (EEA), 2024). Nevertheless, at national and regional levels (including Italy and Emilia Romagna), CE implementation often remains centred on waste management systems and large private actors, reinforcing a narrow interpretation of circularity (Cappellaro et al., 2020; Ghisellini and Ulgiati, 2020).

At the urban level such shortcomings are particularly evident, as cities are often framed as testing grounds for circular approaches. In diverse urban contexts, actors state the goal of “becoming circular” (e.g., Amsterdam, Torino) and of positioning their territory as a “leading circular city” (e.g., Glasgow), developing both broad circular strategies and more targeted circular interventions (Petit-Boix and Leipold, 2018; Santonen et al., 2017 in Kębłowski et al., 2020, 143). Despite its “essentially contested” character (Korhonen et al., 2018) and the lack of evidence for its ecological and socio-economic transformative capacity, the notion of perfect circularity is often taken for granted and reiterated as an ideal, including in urban and metropolitan contexts (Gregson et al., 2015). The circular discourse is adopted by policymakers and consultants in an uncritical, descriptive, and normative manner, and is sometimes conflated with mere “sustainability” (Montenegro Navarro, 2018). The notion of the “circular city” often remains underdeveloped. The lack of context-sensitive methods to co-create locally adapted circular strategies hinder implementation at the territorial level, where CE initiatives may encounter social and spatial tensions (Murray et al., 2017). Existing studies criticize the marginal role afforded to citizens and the emphasis on major urban actors and digital/data driven solutions (Kębłowski et al., 2020; Prendeville et al., 2018). Furthermore, urban CE strategies tend to replicate waste centred and technocentric priorities, struggling to move beyond end of pipe solutions (Gravagnuolo, 2022). Such approaches threaten the transformative potential of CE by neglecting local needs, relational capacities and place based dynamics (Geissdoerfer et al., 2017).

Advancing a more systemic, equitable and context sensitive CE therefore requires expanding innovation beyond technological and industrial processes toward an innovation ecosystem perspective (Granstrand and Holgersson, 2020), involving a broader range of actors, including users, communities and civil society, across production and consumption domains and multiple scales (Kirchherr et al., 2017; Reike et al., 2018). From this viewpoint, the circular economy is shaped not only by technologies and markets but also by territorial features - social relations and institutional arrangements - that, through regional and urban governance, can support the availability of resources and a more balanced spatial distribution of manufacturing. The cycle itself (reuse, repair, recovery and recycling) implicitly relies on spatial proximity between its phases, since sustainability is undermined if materials travel continental distances generating additional externalities. Operationalising a “strong” CE thus implies redistributing production and service activities across the territory rather than focusing solely on technological or market interventions (Arauzo-Carod et al., 2022). Accordingly, research has increasingly been encouraged to examine CE strategies alongside the barriers and opportunities identified by key stakeholders within specific urban and regional contexts (Petit-Boix and Leipold, 2018).

Within this broader transition, the development of new and more diverse circular business models (CBMs) plays a crucial role, as they can reorient actors’ behavior, redefining how value is created, distributed and captured, while contributing to the reduction of environmental impacts through shorter and more localised material loops (Bianchini et al., 2019). Although several CBM frameworks have been proposed, their practical implementation remains challenging and uneven (Geissdoerfer et al., 2018; Lieder and Rashid, 2016; Suárez-Eiroa et al., 2019). Moreover, literature linking CE and entrepreneurship primarily reflects market oriented logics (Suchek et al., 2022), with limited attention to user oriented practices and prevention based strategies (Szabó et al., 2024; Williams, 2019). This gap is particularly evident regarding grassroots circular initiatives. While CE scholarship has increasingly emphasised the need to incorporate social dimensions and actors beyond industrial and policy spheres (Corvellec et al., 2022; Geissdoerfer et al., 2018), empirical research remains scarce. Recent literature encourages future research collecting insights from “alternative organizations that not only focus on how to produce but also on why to produce and who produces” (Hossain et al., 2024). Grassroots innovation, understood as networks of organisations and activists who propose bottom up, sustainability oriented solutions to local challenges (Seyfang and Smith, 2007), offers a useful lens to explore how circularity is (re)interpreted, practised and contested at the urban level (Acheson et al., 2024). However, within the circular economy literature approached from a sociological perspective, limited attention has been paid to the contextual factors and policy conditions that enable or constrain their contribution to circular transitions.

## 3 Methodology

### 3.1 Grassroots circular innovation and living labs: a research approach

The living lab (LL) is defined as a user-centred, open-innovation environment based on a systemic approach to co-creation that integrates research and innovation processes within real-life contexts (Hossain et al., 2019; Leminen et al., 2017a), highlighting everyday practices where stakeholders’ needs and expectations emerge. In this study, the LL functions as a collaborative research device to observe how circular practices emerge, interact and encounter constraints within specific urban configurations. The LL involved local organisations and combined participatory mapping, co-design workshops and qualitative documentation to elicit actors’ practices, resource flows and their interactions with local governance structures. LLs are inherently multi-stakeholder platforms bringing together heterogeneous actors - users, civic organisations, third-sector bodies, companies, researchers and public institutions - who act as resource holders able to mobilise knowledge, skills, material resources and relational capacities (Leminen and Westerlund, 2017b; Nyström et al., 2014; Westerlund and Leminen, 2011; Schaffers and Turkama, 2012). In the Bologna LL, this mobilisation centred on knowledge and skills around reuse, repair and sharing practices and the pooling of local infrastructures and networks.

The critical literature on living labs, however, emphasizes the risk toward a “hollow rhetoric of experimentation”, whereby experimentation is often instrumentalized to satisfy institutional demands for innovation and creativity rather than to meet rigorous methodological standards. Many scholars argue that such initiatives fall short of “deep experimentalism” (after Dewey) and lack a coherent epistemological foundation, limiting their capacity to produce lasting and equitable policy change. Institutional constraints, methodological weaknesses, and a dominant innovation discourse frequently privilege symbolic outputs over substantive transformation, leading living labs to generate short-lived or superficial interventions rather than sustainable reforms (see, e.g., Berger, 2024). In our case, the main concern relates to the project-based approach (ibidem), which risks confining the experiment to the temporal and spatial boundaries of the single project^3^.

From an ecosystem perspective, LLs can be interpreted as circular-economy ecosystems where diverse actors collaborate around flows of material resources, knowledge and socio-economic value (Engez et al., 2021). In the Bologna case, mapping and workshop outputs documented material and knowledge flows (e.g., reuse streams, repair skills exchange, shared service arrangements) that enabled cooperative dynamics supporting reuse, repair, sharing and regeneration (Cuomo, 2022) and provided an analytical lens to observe interaction patterns, coordination mechanisms and systemic barriers to bottom-up circular innovation. The LL was designed to shape shared interpretations, collective learning and common problem framings, which emerged repeatedly in participant reflections and were triangulated through session notes and project deliverables. Methodologically, the LL produced situated knowledge and collective sense-making (Almirall and Wareham, 2011), operating as an intermediary arena between local practices and institutional domains, making visible governance gaps and infrastructural barriers (Amenta et al., 2019; Cuomo et al., 2020). The LL thus provided the opportunity to explore emergent CBMs and the challenges faced by grassroots initiatives when interacting with broader actor ecosystems.

### 3.2 Research design and data organization

The Emilia-Romagna region shows an active involvement in the promotion of CE at both territorial and urban levels. The region established a dedicated law to support the transition from a linear to a circular economy, accompanied by several regulatory frameworks addressing sustainable waste management, renewable energy development, urban regeneration, and the civic awareness through local initiatives promoting the efficient use of resources (Ferraioli et al., 2024). These policy efforts have contributed to the emergence of a favorable ecosystem for circular practices, including reuse centers, community initiatives, and social enterprises.

More specifically, the Municipality of Bologna has committed to reaching climate neutrality by 2030 and is implementing a series of measures aligned with national and European strategies, including interventions on environmental information transparency, climate mitigation and adaption, and civic participatory tools. However, despite this active sustainability agenda, the city still lacks a dedicated strategy for CE (*ibidem*). The combination of these factors makes Bologna a particularly relevant case study for exploring how grassroots initiatives interact with emerging circular economy frameworks and urban sustainability agendas. Within this context, the presence of both established institutional actors and small-scale grassroots organizations creates a dynamic environment in which different interpretations of circularity coexist. Although explicit conflicts are not always visible, tensions may emerge in terms of access to resources and regulatory recognition for alternative practices.

This article presents a qualitative research study conducted within the ECOSISTER project, a large-scale, cross-sectoral initiative aimed at fostering regional ecological transitions through innovation, inclusive engagement, and an ecosystem approach. Within ECOSISTER, Work Package 1 of Spoke 5 focuses on the interplay between circular business model innovation and public policies for sustainable development. Addressing the limited visibility of alternative, place-based and bottom-up forms of circularity within dominant waste- and industry-oriented CE pathways, this study complements other project activities that adopt sectoral and vertical perspectives (e.g., agriculture, manufacturing, tourism).

To this end, the *R-innovare l’Economia Circolare* living lab was designed as participatory research setting to investigate how grassroots initiatives in the metropolitan area of Bologna interpret and enact the circular economy, including the circular strategies and business models they employ, and which barriers, challenges and infrastructural gaps limit their capacity to contribute to urban circular transitions. The living lab ran in Bologna between January and June 2025 and involved 17 organisations and a total of 43 participants across in-person co-design workshops and multiple follow-up and validation sessions. Recruitment combined purposive and snowball sampling, prioritising small-scale, locally embedded initiatives directly engaged in material flows (textiles, food surplus, electronics, furniture, building materials), including informal groups, voluntary associations, social cooperatives, SMEs and start-ups involved in proximity-based circular practices such as reuse, sharing, repair and remanufacturing.

The process unfolded through interconnected phases of mapping and scouting, participatory activities to surface CBM practices, needs and barriers, co-design sessions to develop shared interpretations and local policy suggestions, and a final validation and dissemination phase. A professional facilitation team supported engagement activities, and an advisory board of researchers, local public agency representatives and business practitioners supported scientific robustness, policy relevance and strategic orientation. Empirical materials include workshop audio recordings and facilitator notes, participant surveys (19 responses from representatives of local organisations involved in the living lab), participatory maps, clustering exercises, group discussions using design-thinking tools, project documentation and aggregated deliverables such as a manifesto presenting directions to support grassroots circular business models, which was publicly discussed with a broader stakeholder audience.

Qualitative data, primarily drawn from workshops, group discussions and facilitation materials and complemented by surveys collecting organisational information, were analysed through an iterative process of thematic coding and interpretation to identify recurring practices, shared barriers, governance challenges and CBM archetypes. Participants in the LL were asked to identify their circular strategies using the R-strategies framework (R0-R9) (Reike et al., 2018). To ensure alignment between declared strategies and actual activities, a qualitative cross-check was conducted for each case. The R-strategies were grouped into three macro-categories: (i) user choices and shortest-loop strategies (R0–R3), focused on consumer engagement, waste reduction and reuse; (ii) product upgrading and medium-long loops (R4–R6), centred on refurbishment and repurposing; and (iii) material downcycling and recovery, long-loop strategies (R7–R9), focused on material flow management and transformation. The CBM archetypes were derived through theory-driven coding using established frameworks (Bianchini et al., 2019; Bocken et al., 2016; Ellen MacArthur Foundation, 2015; Reike et al., 2018; Woldeyes et al., 2022), such as the most frequently adopted RESOLVE^4^, and validated in participatory sessions with workshop participants and the advisory board. The Bologna living lab generated three main outputs informing the results section: profiles of grassroots circular organisations, mappings of circular practices and shared CBMs, and collectively identified barriers and challenges.

## 4 Results

### 4.1 Involved grassroot organizations profile

The 17 engaged organisations operate at the intersection of environmental sustainability, social innovation and proximity services (proximity here denotes the spatial closeness of actors, resources and practices within a given local environment). Most participants were third-sector organisations: seven social cooperatives, four associations for social promotion, one voluntary organisation and one associative network. The remaining four comprised SMEs and start-ups.

Participating organisations implement local CE practices such as reuse, repair, sharing and material recovery, often prioritising waste prevention over waste management. Environmental objectives frequently coexist with social aims: many activities are driven by inclusion of vulnerable groups, social equity, anti-marginalisation and territorial regeneration. Examples include cooperatives employing disadvantaged people in textile collection and sorting, artisan labs transforming glass and wood production leftovers into new goods, and volunteering associations collecting unused sports equipment for redistribution. The full list of participating organisations, with main activities and focus, is reported in Table 1.

**Table 1:** List of organizations involved in the living lab, main activities and sectors.

ID	General description	Practices performed (not exhaustive)	Main sector/domain
1	NGO focused on reducing environmental impact of public events and rising awareness on responsible resources use.	Reusable tableware lending service	Events/services
2	Regenerated peri-urban space promoting education, sociality and self-sufficiency.	Phytodepuration, composting, water reuse	Urban regeneration
3	Social and charity project promoting recycling to foster social and labor inclusion of fragile.	Collection and resale of recyclable materials and textiles	Waste/textiles/inclusion
4	SME designing aquaponic systems for sustainable food production in urban spaces.	Closed-loop integration of fish farming and plant growth, urban space rethinking	Urban agriculture/urban regeneration
5	Community Supported Agriculture (CSA) based cooperative promoting food sovereignty and agroecology	Agroforestry on public land, local food self-production, education	Agriculture/food
6	Association promoting an anti-disposable culture and circularity awareness.	Plastic upcycling labs, reusable cups for events, education and public events	Education/events
7	App enabling sharing and reuse among neighbors and small shops.	Peer-to-peer reuse of used goods, digital platform for reusable and unsold products	Digital/community sharing
8	Urban beekeeping initiative supporting biodiversity and education.	Urban space regeneration, Environmental biomonitoring, education	Biodiversity/education
9	Ethical fashion laboratory employing refugees through upcycling leather leftovers from main manufacturers.	Upcycling of leather leftovers and scraps	Fashion/inclusion/arts & crafts
10	Social bike repair shop promoting sustainable mobility and job inclusion.	Bike repair, re-manufacture of abandoned bikes	Mobility/inclusion
11	Cultural association supporting food and goods redistribution for disadvantaged people and social initiatives.	Community fridge, clothing and food reuse	Social welfare/culture
12	Cooperative for green maintenance, waste management, and composting employing disadvantaged people.	Local composting, biofuel production, waste collection and sorting	Waste/services
13	Social enterprise upcycling glass and other materials through inclusive artisan labs.	Creative reuse of recovered glass, artisan upcycling	Inclusion/arts & crafts
14	Business cultivating edible mushrooms on organic waste like coffee grounds from restaurants and local businesses	Urban farming on biowaste	Food/urban agriculture
15	Social coop fighting marginalization and supporting vulnerable people with reuse-based projects.	Material collection and selection, reuse workshops and shops for labor inclusion, furniture and assets reuse	Inclusion/welfare
16	Volunteer-based project redistributing repaired sports equipment for social inclusion in sport	Repair and donation of used sport gear	Sport/inclusion
17	Municipal reuse center operated by a social coop with free access for citizens	Collection and redistribution of second-hand goods	Waste/public services

Geographical data on operational sites show that all organisations are based within the metropolitan city of Bologna, although several also operate across urban and peri-urban areas beyond municipal boundaries. The actors involved reflect a strong orientation toward social objectives combined with entrepreneurship, local embeddedness and the capacity to mobilise both broad and proximity-based networks, including marginalized groups, migrants, and victims of trafficking.

### 4.2 Circular practices and emerging circular business models from grassroot actors in Bologna

**Figure 1:**
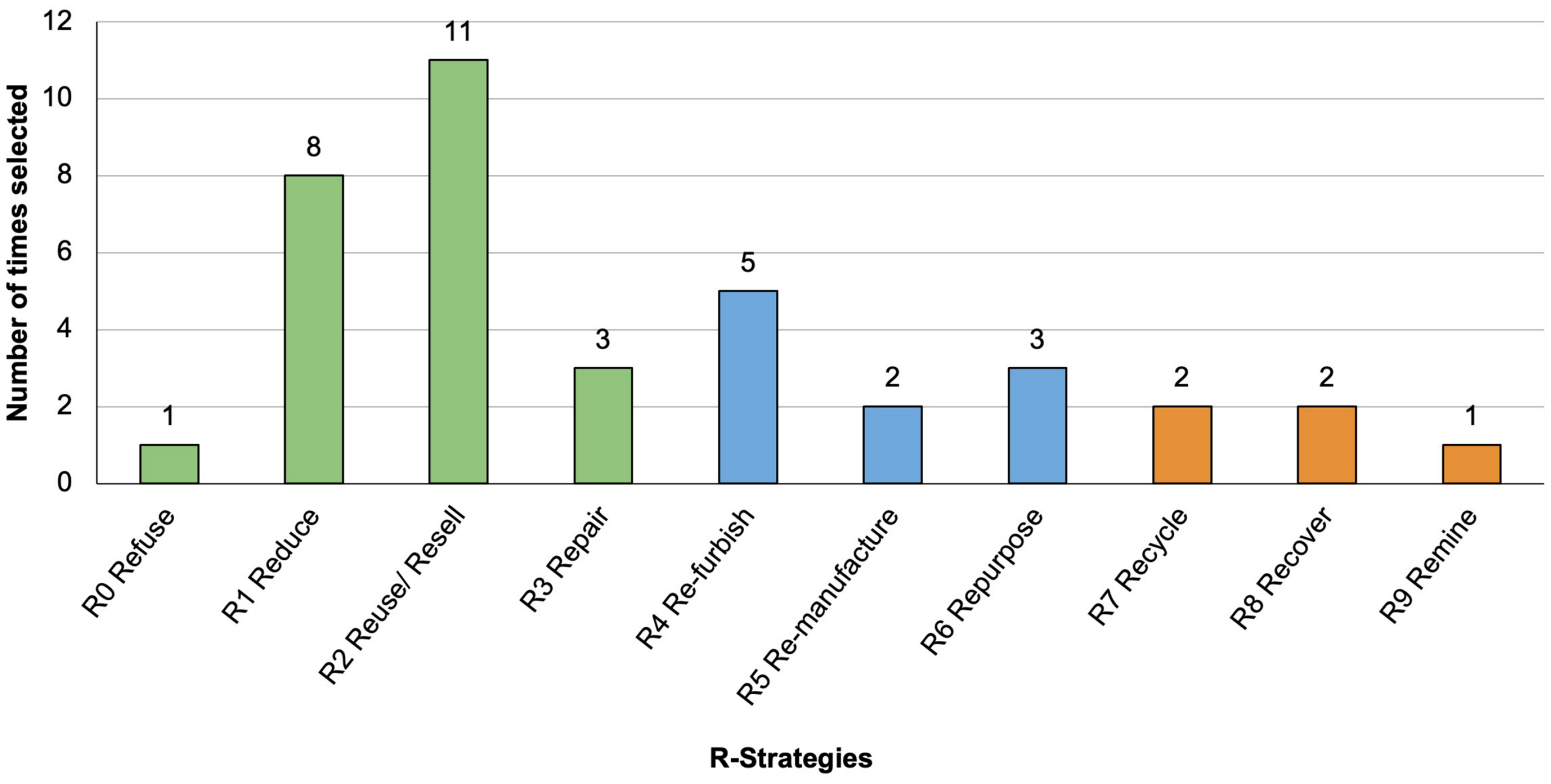
R-strategies selected by grassroots organizations (shortest loops R0–R3, medium long loops R4–R6, long loops R7–R9).

Figure 1 shows circular practices implemented by participating organizations, clustered by their focus on the creation of short, medium or long circular loops. The most frequently selected strategies were R2 - Reuse/Resell (11 organisations) and R1 - Reduce (8), indicating a strong emphasis on waste prevention and extending product lifespan. Strategies involving more technical stages, such as R4 - Refurbish, R3 - Repair and R6 - Repurpose, were less selected (5, 3 and 3 respectively), but indicate specialised capacities within the network. The least adopted strategies were R0 - Refuse/Replace and R9 - Remine, each selected by only one organisation: R0 by an actor advocating zero-waste practices and refusal of single-use approaches, and R9 by a social cooperative running a material recovery facility. These limited occurrences reflect the difficulty for grassroots actors to operate at the design or industrial scale of production systems.

This preliminary exploration suggests a multidimensional yet uneven engagement with CE practices across the product life cycle. The mapping indicates a diversity of approaches, with a strong emphasis on upstream prevention and reuse (shortest loops) while, to a lesser extent, contributing to product upgrading and downstream recovery (medium-long loops). Several initiatives combine technical practices (repair, remanufacturing) with social or artistic engagement, highlighting the potential need for policy frameworks supporting heterogeneous, context-specific circular models.

During in-person workshops, organisations - supported by expert facilitators and researchers - self-clustered into groups based on affinity with specific CBM archetypes, forming relatively homogeneous teams to explore common barriers, needs and peer-to-peer solutions. Some participants aligned clearly with a single model, while others positioned between multiple archetypes. Confusion often arose around distinctions such as reuse versus recycling or material reuse versus waste recovery, and regulatory ambiguities complicated classification for actors working with secondary materials outside formal waste regimes. For these actors, the risk of being perceived as operating unauthorised waste streams - exposing them to inspections, sanctions or reputational harm - emerged as a recurrent concern. Facilitated discussions with researchers helped clarify these issues and supported participants in clustering within the most coherent models.

The process identified three context-sensitive CBMs: (i) Reuse/Repair/Sharing; (ii) Regeneration; and (iii) Recovery and Recycling of Materials and Scraps (see Table 2 for descriptions and examples). Despite differences, these models share cross-cutting features, including strong local embeddedness and a twofold value proposition combining environmental outcomes with social and economic benefits. The observed diversity indicates how CE principles are translated into place-sensitive practices and suggests the need for more differentiated, inclusive policy frameworks supporting a broader spectrum of circular experimentation.

**Table 2:** Emerging circular business models from grassroots organizations.

Circular business model (CBMs)	Short description	Example of practices
1. Reuse, repair & sharing (*n* = 7)	Models that extend the lifespan of products through reuse, maintenance, repair, and sharing of goods and services, keeping items in circulation and slowing down consumption.	Reuse centers and shops, repair workshops, digital marketplaces, second-hand redistribution, sport equipment exchange.
2. Regeneration (*n* = 4)	Practices and models that promote the use of renewable resources, the management and closure of biological and water cycles, and the restoration and enhancement of urban and rural ecosystems.	Regeneration of degraded spaces, sustainable water systems (e.g., phytodepuration), agroforestry, composting, peri-urban revitalization, community-based activities.
3. Recovery of materials, scraps and recycling (*n* = 6)	Strategies that valorise post-consumption and discarded materials (sometimes waste) through upcycling, recycling and transformation into new resources or goods with added value.	Upcycled leather goods, creative glass reuse, inclusive artisan labs, local composting, biomass valorisation, food redistribution, mushroom farming.

### 4.3 Understanding the infrastructural gaps. Barriers and needs for CBMs implementation

Following group creation, participants at each table were invited to identify and discuss common obstacles and needs related to CBM implementation. Main insights are reported in Table 3.

**Table 3:** Key challenges and needs of identified CBMs.

Model	Challenge area	Key issues/needs
Reuse, repair & sharing	Access to spaces and infrastructure	Lack of physical spaces for storage and sharing; need for dedicated areas (e.g., shared warehouses, donation spots); difficulty maintaining headquarters and equipment (e.g., dish libraries).
	Economic sustainability and incentives	Difficulty ensuring economic viability of reuse/sharing initiatives; need for fiscal incentives (e.g., VAT and waste tax reduction); application of EPR^1^; improved access to funding and public support.
	Regulation and institutional recognition	Regulatory context-specific constraints (e.g., ban on reusing spare parts); lack of clear frameworks for redistribution of used goods; weak institutional recognition of social value; need for policies supporting local actors over external operators.
	Culture and demand	Low and unstable demand for second-hand/shared goods; competition with new products; poor quality of donated items; limited awareness of cost-effectiveness and environmental benefits; misinterpretation of free sharing systems.
	Networking and collaboration	Need to connect sharing actors; difficulties in identifying reliable partners; importance of exchanging practices and amplifying impact through collaboration.
	Communication and digital innovation	Need for better communication of sharing services, their purpose and value; need for digital tools to improve visibility, logistics, and access (e.g., virtual platforms, matchmaking tools);
Regeneration	Access to common goods and spaces	Difficulty in accessing and regenerating underused spaces (public and private); lack of connections between owners and users; dominant commercial interests over social/community use.
	Bureaucracy and regulations	Administrative work and delays; unclear or non-existent regulations for innovative practices (e.g., phytodepuration); need for specific protocols ensuring recognition and safety.
	Economic sustainability and funding	High initial investment vs. long-term sustainability; limited access to funding; need for stronger connections between institutions and social projects.
	Water resource management	Need to reduce water waste and promote reuse; barriers to implementation of wastewater reuse; lack of incentives for sustainable water management.
	Networking and collaboration	Need for platforms to match available spaces with potential users; importance of cooperation among projects for knowledge/resource exchange and strengthening horizontal networks to increased collective impact.
Recovery of materials, scraps and recycling	Market issues and economic sustainability	High recycling/upcycling costs; low and unstable market demand for secondary raw materials (e.g., compost, recovered leather); distorted price mechanisms make disposal cheaper than recovery.
	Material management and closed loops	Fragmented and inefficient logistics; technical and legal barriers to product reuse; poor quality in material sorting; lack of integrated local recovery chains.
	Regulations and incentives	Need for fiscal incentives to push companies taking back of recovered products; support for bans on destruction of unsold goods; extension of EPR to more product categories.
	Awareness and education	Need for public education on sorting and recycling practices; raise awareness on the environmental risks of superficial circular solutions; encourage responsible consumer behavior.
	Cross-sector collaboration	Need to foster synergies among businesses for creating local circular loops; connect waste donors with users; strengthen collaboration networks to scale recovery initiatives.

1 EPE, extended producer responsibility.

A series of transversal challenges emerged across the different realities involved. These obstacles slow the development of existing initiatives and limit their long-term consolidation but also highlight shared needs that could be addressed through targeted strategies and policies.

One recurrent issue concerns access to space and infrastructure. Organisations supporting reuse, sharing and regeneration struggle to find adequate places for their activities warehouses for storage, venues for sharing goods or urban spaces to be regenerated. Management and maintenance of these environments also pose obstacles, especially in the absence of constant use and sustainable economic models. The lack of connections between owners and potential users further complicates viable solutions.

Economic sustainability is another critical issue. Many initiatives generate environmental and social benefits, but without tax incentives, concessions on operating costs (such as reductions in VAT or TARI^5^) and access to public funding, they cannot sustain themselves in the long term. Moreover, demand for remanufactured products and recycled materials often remains insufficient to cover treatment and recovery costs, making recycling and reuse less competitive than traditional disposal.

Regulatory and bureaucratic barriers further complicate the work of those experimenting with sharing, remanufacturing and recovery models. The lack of clear regulations on material recovery and the redistribution of used goods creates uncertainty, while local restrictions (e.g., bans on the recovery of spare parts or the absence of protocols for phytodepuration^6^) limit innovative solutions. Many participants stressed the need for territorial policies that incentivise and valorise local realities, preventing external operators from gaining competitive advantage over actors already embedded in the territory.

Networking and collaboration emerged as another central theme. While willingness to share good practices was evident, organisations reported difficulties in finding reliable partners for redistribution of goods and materials. The lack of tools and platforms facilitating networking limits the possibility of creating integrated supply chains on a local scale.

Strengthening the culture of reuse and raising awareness - both at institutional level and among citizens - was also emphasised. The value of CE is not yet fully recognised: many organisations reported a lack of institutional awareness regarding the economic and environmental benefits of their activities. Consumers often struggle to understand the value of reuse versus disposability, and some sharing projects encounter opportunistic behavior from users.

Finally, participants highlighted the importance of communication tools and digital innovation to support visibility and functioning of initiatives. Creating virtual showcases for exchange of goods and materials, improving communication with the public and institutions, and developing tools for logistics and matchmaking between supply and demand are considered key steps to make circular practices more accessible and sustainable.

These surfaced challenges emphasise the importance of collaboration, cross-sectoral coordination, and the creation of enabling environments such as support infrastructure, accessible spaces, appropriate incentive schemes and inclusive decision-making processes. They highlight the need to strengthen socio-economic factors that sustain circular practices, suggesting that the success of CBMs may depend as much on supportive ecosystems and cultural shifts as on technological advancements.

## 5 Discussion

### 5.1 Reconceptualizing circularity: care, prevention and community practices

Drawing on the “R-innovare l’Economia Circolare” living lab in Bologna, this study provides empirical insights into how circularity is locally shaped and enacted at the urban scale. The participatory approach surfaced context-specific tensions, barriers and infrastructural gaps affecting grassroots CBMs. Evidence from Bologna suggests that, for many engaged organisations, circularity functions less as an end and more as a means to advance broader systemic goals - social inclusion, territorial regeneration and community well-being - and that circular practices operate as place-based responses to societal challenges beyond narrow resource-efficiency framings.

A distinctive feature of these initiatives is their emphasis on waste prevention and practices entailing direct interactions with citizens, largely situated in the use phase of products where habits, everyday routines and locally embedded cultural norms shape consumption and material use. By engaging people at this level, grassroots initiatives and organizations working with marginalised groups can create opportunities to promote fairer and more inclusive circular transitions.

From this vantage point, grassroots circular models often struggle to align with mainstream interpretations of circularity, particularly those in CBM literature oriented to business and industrial sectors that may underrepresent urban, community-driven dynamics. Widely used frameworks tend to emphasise production over consumption, giving limited attention to local context and overlooking scale and localization, dimensions found central when circularity is enacted by grassroots actors.

In line with calls to investigate underexplored CBMs, our findings indicate that sharing, maintenance, collection, upgrading and redistribution emerge as core components of grassroots circularity. Tensions observed in Bologna reflect a broader gap in CE and CBM scholarship: grassroots, public and hybrid initiatives, often motivated by social purpose rather than profit, remain analytically marginal despite their growing relevance for urban circular practices and socio-ecological transitions. Several organisations resisted being framed as “businesses,” raising questions about whether existing CBM frameworks capture their motivations and value propositions. These findings align with research on urban experimental niches (see, e.g., Savini and Bertolini, 2019), understood as emerging social practices (niches) that may be disruptive within their normative and physical contexts. When politically framed and subject to deliberate governance, niches can be identified as sites of potential institutional change. Such change does not occur spontaneously but through a political process that involves recognition, the definition of a trajectory (either conforming to or challenging the existing order), and the organisation and mobilisation of resources to coordinate, regulate, or nurture the practice, thereby actively shaping the social norms and physical spaces in which it operates.

Recent contributions (see, e.g., Miranda de Souza et al., 2026) that conceptualise community-oriented reuse organisations point to the need to expand CE frameworks and policy instruments to better recognise multidimensional, community-based pathways through which circularity is enacted in cities. Crucially, circularity as practised by grassroots actors incorporates social capital - people, workers and community relations - into the flows CE seeks to sustain and regenerate. Initiatives that reintegrate marginalised groups via education, skills development and artisanal training (described by participants as a “restoration of dignity”) indicate that the social dimension of CE extends beyond job creation to include strengthening community bonds, preserving local knowledge and providing welfare-oriented services.

These interpretations resonate with systemic visions of CE (reformist and transformational circular society discourses) and recent conceptualisations of CE in cities and territories, extending circularity beyond material flows and recycling to include intangible assets - culture, identity and social capital - and emphasise regeneration of human capital alongside environmental safeguards. Overall, these insights underscore the importance of including underexplored voices in the framing and implementation of CE to better align circular initiatives with intended societal benefits.

### 5.2 Enabling bottom-up circular innovation: governance gaps and infrastructural needs

While grassroots circular initiatives broaden the understanding of circularity at the urban scale, their implementation remains constrained by infrastructural and governance challenges. The organisations involved vary widely in structure, resources, and operational capacity, from formal social cooperatives to less structured volunteering associations and local movements; in some cases, hybrid public–third sector forms are involved. This diversity suggests that circular transitions engage heterogeneous stakeholders with uneven capacities, calling for differentiated approaches suitable to engage different segments of society (Kirchherr et al., 2023) and appropriate tools for stakeholder engagement (Innella et al., 2024). Although public institutions increasingly aim to combine urban regeneration with territorial economic development, our findings suggest that existing governance arrangements only partially align with the needs of bottom-up circular innovation. Experimental approaches to policy development and co-production are being mobilised as tools to manage socio-ecological transitions (Cuomo, 2022; Nesti, 2018), yet their application remains uneven and limited in scope. In this context, the Bologna living lab functioned as a temporary space for participatory engagement, mutual learning, and horizontal collaboration. Its user-centred, open-innovation setting enabled identification and analysis of grassroots CBMs operating within the territory. Through co-creation processes, participants, researchers, and practitioners surfaced implementation barriers from a systemic perspective, embracing collective sense-making and shared ownership of issues and solutions. However, this experimental space was limited in time and scope, circumscribed to a specific project and territorial area, raising questions about its capacity to generate long-term impacts or become institutionalised. The challenges reported by grassroots actors point to structural gaps within current socio-technical infrastructures that constrain consolidation and scaling of bottom-up circular initiatives^7^. A first critical gap concerns limited institutional recognition of the social and environmental value generated by grassroots circular initiatives, particularly those oriented toward reuse, repair, and regeneration, which often fall outside dominant recycling and industry-focused frames. This lack of legitimisation also reflects difficulties in assessing circular impacts beyond economic and material dimensions. As noted by Ranta et al. (2018), limited institutional legitimacy outside recycling strategies remains a persistent barrier to CE implementation, resulting in uneven territorial support and dependence on the sensitivity of individual administrations. Actors working with recovered materials outside formal waste management channels fear being perceived as handlers of unauthorised waste, with attendant risks of inspections, sanctions, or reputational damage; such concerns evoke “blame avoidance” dynamics (Weaver, 1986; Howlett, 2012) common in public policy processes and, particularly where automated decision-making mechanisms are employed, undermine the reuse opportunities offered by actor networks. A second governance challenge relates to the position of grassroots circular practices at the intersection of multiple policy domains. These initiatives frequently blur boundaries between CE and other paradigms such as sharing economy (Sposato et al., 2017), social and solidarity circular economy (Monciardini et al., 2024), eco-welfare (Carrosio and Cogliati Dezza, 2025). While this hybridity can generate innovation, it also exposes initiatives to fragmented governance. In this context, the role of facilitators and intermediary actors becomes also particularly relevant, as they can help connect different policy domains and support more integrated approaches (Domorenok et al., 2021). Supporting bottom-up circular innovation thus requires governance frameworks capable of integrating these paradigms within more cohesive strategies. Finally, participants repeatedly emphasised the absence of “innovation environments” (Montanari and Mizzau, 2016): spaces for open discussion and collaborative planning on CE within their local networks. This underscores the central role of proximity - spatial closeness, shared practices and the capacity to interact - in enabling emergence and development of CE initiatives (Chembessi et al., 2024).

Overall, insights from the Bologna case indicate that challenges faced by grassroots circular initiatives are not organization-specific but embedded in broader configurations that continue to favour linear, sectoral and recycling-oriented approaches to circularity. Addressing these dynamics requires interventions targeting institutional recognition, conflicts between policy domains, and the orchestration of appropriate innovation environments that support the co-creation of sustainable, place-based circular transitions.

Nevertheless, the generalizability of the finding remains limited, as the process engaged a relatively small sample operating within a specific geographic and temporal context. Despite efforts to ensure inclusiveness, participation was temporally and spatially constrained and may have excluded relevant voices. The co-identified barriers reflect context-specific challenges that may not directly apply to other territories and sectors.

## 6 Conclusions and future directions

While the circular economy (CE) remains a central framework for sustainable development and urban regeneration, its prevailing waste-centred, technocentric implementation limits its transformative potential. Moving beyond recycling toward prevention, reuse and socially embedded practices is essential to realise the socio-ecological promises of circular transitions.

This study explored alternative pathways to enact circularity through a participatory living lab in Bologna, engaging 17 grassroots and civic organisations. Focusing on bottom-up CBMs and the barriers they face in consolidating and scaling activities, the research aimed to surface interpretations and practices of circularity that diverge from mainstream, industry-led approaches.

Findings indicate that grassroots initiatives can broaden dominant CE narratives by integrating solidarity, reciprocity and care, thereby enriching CE’s social dimension. Circularity as practised by these actors extends beyond material flows to incorporate social capital and community relations into the “loops to manage,” supporting calls for more holistic, human-centred CE approaches. Inclusive CE implementation that emphasises prevention-oriented strategies may yield greater socio-ecological benefits than a narrow focus on waste management and recycling.

Working within local ecosystems introduces complexity but appears essential to unlock context-sensitive innovation and support more just and inclusive circular transitions. The identified barriers point to structural misalignments between grassroots practices and existing economic, financial and policy infrastructures, indicating the need to: (i) improve institutional recognition of grassroots value; (ii) redesign incentive schemes to include social and preventive outcomes; and (iii) enhance cross-policy coordination to better support bottom-up ecological and social innovation.

Future research could: (a) conduct comparative studies across territorial settings to distinguish place-specific from structural factors; (b) assess how competing CE narratives (industrial vs. grassroots) affect implementation outcomes, material and social resource allocation, policy support and visibility; and (c) investigate whether and under what conditions insights from participatory approaches (e.g., living labs) can influence public decision-making and policy design.

## Data Availability

The raw data supporting the conclusions of this article will be made available by the authors, without undue reservation.

## Footnotes

1 The Metropolitan City of Bologna is an Italian metropolitan city in Emilia-Romagna with 1,024,063 inhabitants. It comprises 55 municipalities across an area of 3,825 km.

2 Ecosystem for Sustainable Transition in Emilia-Romagna ECOSITER, funded by National Recovery and Resilience Plan PNRR - Mission 4 - Component 2. ECS00000033. Spoke N. 5 - Circular economy and blue economy.

3 Efforts are nonetheless underway to identify pathways for further development beyond the specific project framework.

4 REgenerate, Share, Optimise, Loop, Virtualise, and Exchange (Ellen MacArthur Foundation, 2015).

5 TARI is the italian municipal waste tax.

6 Natural supported treatment system for domestic, agricultural and, in some cases, industrial wastewater, by means of self-purification process typical of aquatic environments and wetlands.

7 Bottom-up experiment scalability is shaped by local institutions. Investigating grassroots smart innovations, Raven et al. (2019) identify three logics: (1) Ecosystem - evolutionary scaling from many small pilots facilitated by flexible public-private coalitions; (2) Social-learning - scaling via mutual understanding and cooperation among state, firms and universities, guided by municipal investment; (3) Managerial/central-control - hierarchical scaling led by government experts who select and disseminate best practices.
